# Genotype by environment interaction for 450-day weight of Nelore cattle analyzed by reaction norm models

**DOI:** 10.1590/S1415-47572009005000027

**Published:** 2009-06-01

**Authors:** Newton T. Pégolo, Henrique N. Oliveira, Lúcia G. Albuquerque, Luiz Antonio F. Bezerra, Raysildo B. Lôbo

**Affiliations:** 1 Departamento de Genética, Faculdade de Medicina de Ribeirão Preto, Universidade de São Paulo, Ribeirão Preto, SPBrazil; 2 Departamento de Melhoramento e Nutrição Animal, Faculdade de Medicina Veterinária e Zootecnia, Universidade Estadual Paulista Júlio de Mesquita Filho, Botucatu, SPBrazil; 3 Departamento de Zootecnia, Faculdade de Ciências Agrárias e Veterinárias, Universidade Estadual Paulista Júlio de Mesquita Filho, Jaboticabal, SPBrazil

**Keywords:** growth, genotype by environment interaction, plasticity, random regression, robustness

## Abstract

Genotype by environment interactions (GEI) have attracted increasing attention in tropical breeding programs because of the variety of production systems involved. In this work, we assessed GEI in 450-day adjusted weight (W450) Nelore cattle from 366 Brazilian herds by comparing traditional univariate single-environment model analysis (UM) and random regression first order reaction norm models for six environmental variables: standard deviations of herd-year (RRMw) and herd-year-season-management (RRMw-m) groups for mean W450, standard deviations of herd-year (RRMg) and herd-year-season-management (RRMg-m) groups adjusted for 365-450 days weight gain (G450) averages, and two iterative algorithms using herd-year-season-management group solution estimates from a first RRMw-m and RRMg-m analysis (RRMITw-m and RRMITg-m, respectively). The RRM results showed similar tendencies in the variance components and heritability estimates along environmental gradient. Some of the variation among RRM estimates may have been related to the precision of the predictor and to correlations between environmental variables and the likely components of the weight trait. GEI, which was assessed by estimating the genetic correlation surfaces, had values < 0.5 between extreme environments in all models. Regression analyses showed that the correlation between the expected progeny differences for UM and the corresponding differences estimated by RRM was higher in intermediate and favorable environments than in unfavorable environments (p < 0.0001).

## Introduction

Genotype by environment interactions (GEI) occur when the genotype responds differently to changes in the environment (Kolmodin *et al.*, 2002). In recent years, GEI effects have received increased interest because breeding programs tend to be more internationally oriented (Mulder and Bijma, 2005). In addition, the development of molecular genetics has revealed astonishing aspects of epigenetic and major gene by gene and gene by environment interactions (Lewontin, 1998; Schlichting and Pigliucci, 1998) that have revolutionized various genetic concepts (El Hani, 2007). These developments suggest that traditional quantitative genetic models may be underestimating GEI and indicate the need of more precise models for these analyses.

Several studies have examined the importance of GEI in different traits in beef cattle. Most of these studies have revealed strong genetic correlations among different regions or countries, indicating an absence of significant GEI (De Mattos *et al.*, 2000; Johnston *et al.*, 2003). Other studies that have shown important GEI could be questioned because they were local studies and the small number of data used was often a limitation (Bolton *et al.*, 1987; Nobre *et al.*, 1988). In parallel with these investigations, progress in statistical methodology has produced different models and random regression has become increasingly important in longitudinal data analyses. This approach allows genetic parameters to be estimated from repeated stochastic data along a longitudinal variable (Kirkpatrick and Heckman, 1989; Meyer, 1998). The application of these models to growth and lactation curves using the variable “time” in the longitudinal axis resulted in more precise estimates in different phases of lactation (Veerkamp and Thompson, 1999) and growth (Albuquerque and Meyer, 2001). More recently, random regression has been applied to the analysis of longitudinal environmental variables, with a reaction norm concept (De Jong and Bijma, 2002; Kolmodin *et al.*, 2002), based on the set of phenotypes that can be produced by an individual genotype exposed to different environmental conditions (Schmalhausen, 1949). Some evolutionary studies have introduced the term “adaptive” when assessing the value of genetic predictions (Schlichting and Pigliucci, 1998; Sarkar, 1999). Reports describing the use of reaction norms have become more frequent (Fikse *et al.* 2003; Kolmodin *et al.*, 2004). In these studies, the environmental variable is considered to be continuous and can be defined by the proper dataset, thereby avoiding artificial environmental definitions such as national or political barriers. Since genetic parameters are estimated on an environmental gradient, GEI can be identified more precisely based on the genetic correlations between different points on the environmental axis or by the non-parallelism in the estimates of adaptive reaction norms. Environment descriptors and data structure can influence these results, as shown by Fikse *et al.* (2003), Kolmodin *et al.* (2004) and Calus *et al.* (2004).

The aim of this work was to assess the importance of GEI in the 450-day adjusted weights of Nelore cattle by using random regression models and a reaction norm approach. We also evaluated the usefulness of different variables as environment descriptors.

**Figure 1:**
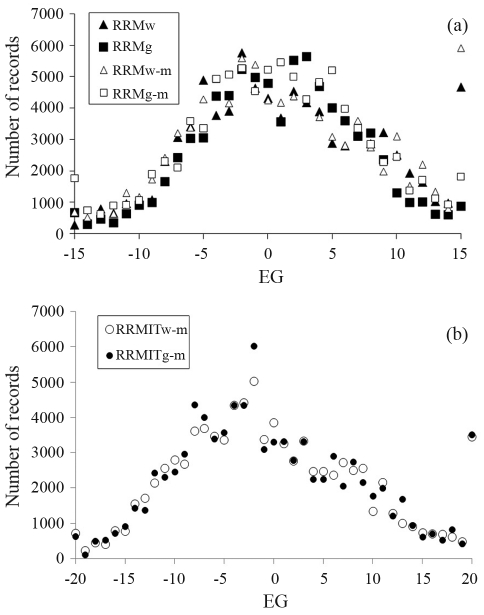
Number of records analyzed in each environmental group for RRMw, RRMg, RRMw-m and RRMg-m (a) and RRMITw-m and RRMITg-m (b).

**Figure 2:**
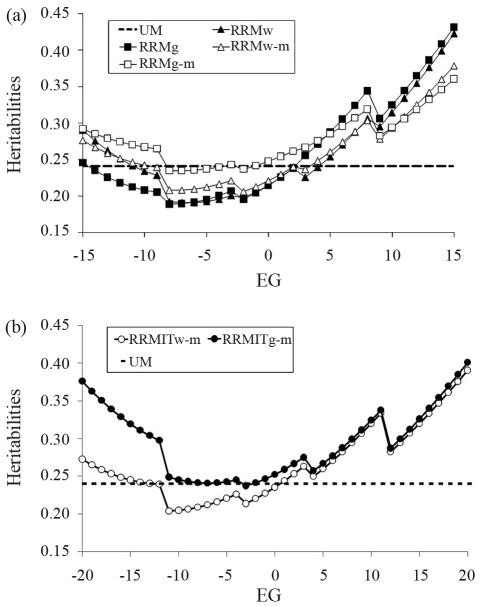
Heritability estimates along environmental group (EG) for UM, RRMw, RRMg, RRMw-m and RRMw-g (a) and UM, RRMITw-m and RRMITg-m (b).

**Figure 3:**
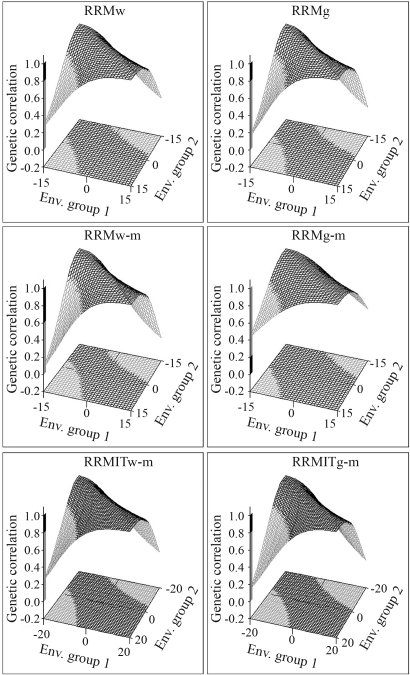
Surfaces of genetic correlation estimates across environmental groups in different random regression models (RRMg, RRMw, RRMw-m, RRMw-g, RRMITw-g and RRMITg-m). The black part of the surface shows r_g_ > 0.8 and the grey part shows r_g_ < 0.8.

## Material and Methods

Data were collected from 366 Brazilian herds by the ANCP (Associação Nacional de Criadores e Pesquisadores) as part of a program for genetic improvement of the Nelore breed. The original dataset consisted of 234,963 adjusted weights for 360 and 450 days (W365 and W450) and weight gain between 365 and 450 days (G450) for Nelore cattle born from 1974 to 2006. The relationship matrix was modified to a sire model because of a constraint of the analysis since it was impossible to expose the same animal to different environments during the same developmental phase. Contemporary groups (CGs) were defined by using information on sex, year, farm, management and calving season; CGs with less than six individuals were excluded.

W450 was studied in seven different models: one univariate single-environment model analysis (UM) and six random regression model analyses (RRMs). The RRM differed only in their environmental descriptor. These were calculated using W450 or G450 contemporary group averages standardized to a mean of zero and an SD of one. The standardized values were then multiplied by ten and the environmental groups (EG) were obtained by considering only the integer part of those values. The integer format is a convenience for subsequent software analyses. In the first and second RRMs, the EGs were based, respectively, on the average W450 (RRMw) and the average G450 (RRMg) of farm-year groups. In the third and fourth RRMs, the EGs were based, respectively, on the average W450 (RRMw-m) and the average G450 (RRMg-m) of farm-year-season-management groups. As management has an implicit sex factor, the records were separated according to sex, and after definition of the environmental groups as standardized W450 averages, the data of the different sex groups were merged by EGs. EG values below -15 were considered in EG = -15 (bottom limit) and those above +15, in EG = +15 (upper limit) (as shown in Figure 1a). The fifth random regression model (RRMITw-m) used an iterative algorithm to define the EGs. In the first iteration, the data were analyzed using RRMw-m and its fixed effect (CG) solutions were used to position records on the respective EG for the subsequent analysis. Since this first iteration resulted in a wide data distribution along the environmental gradient the EG limits were changed to -20 (bottom limit) and +20 (upper limit) from the second to the final iteration (Figure 1b). The process was stopped when the correlation between the EG positions in the previous and present analyses was > 0.999. This convergence was reached after three iterations, in a manner similar to the simulated data used by Calus *et al.* (2004). This process tries to avoid bias resulting from the non-random use of sires or a low number of animals in some herds. The last random regression model (RRMITg-m) used G450-based EGs in the first iteration and repeated the RRMITw-m iterative process.

The EGs were defined using the complete dataset, but additional restrictions were added for parameter estimation. In this case, sires were excluded if (1) they had < 100 progeny weights and (2) the progeny weight distribution along the environmental gradient was < 20 EG units (before the first iteration in RRMITw-m and RRMITg-m). After application of these two criteria, CGs with less than five records were removed. These restrictions affected data differently in the different models and resulted in different numbers of sires and records for the analyses. The UM estimates were based on RRMw data.

(Co)variances of random regression coefficients were estimated by REML using version 3.0ß of the DFREML package (Meyer, 1988). The DXMRR subroutine in the program allowed estimation of the heterogeneous residual variance in five classes. Estimates were obtained by using Powell, Simplex and AI-REML algorithms, thereby avoiding problems with "derivative-free" possible local max estimates. The general model can be represented as follows:



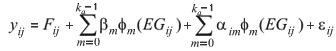


where *y_ij_* is the *j^th^* progeny's W450 or G450 from the *i^th^* animal and *EG_ij_* is the environmental group of the *j^th^* progeny of *i^th^* sire (from -15 to +15 in non-iterative models and -20 to +20 in iterative models), ø*_m_*_(*EGij*)_ is the *m^th^* Legendre polynomial on environmental group, *F_ij_* is the CG fixed effect, ß*_m_* is the fixed regression coefficient to model the population mean (defined only in non-iterative models), a*_m_* is the random regression coefficient for a direct genetic effect, *k_a_* denotes the corresponding orders of fit (one in UM and two for RRMw, RRMg, RRMw-m, RRMg-m, RRMITw-m and RRMITg-m) and e*_ij_* is the error effect associated with the pre-defined classes *p* that have homogeneous variances.

In matrix notation:

y = *X*ß + *Zs* + e

where



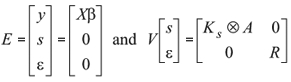


with y being the vector of observations, ß the vector of fixed effect attributable to contemporary groups (including *F_ij_* and ß*_m_*), *s* the vector of sire random coefficients, *X*, *Z* the corresponding incidence matrices, and e the vector of residuals. *K_s_* is the matrix of coefficients of the covariance function for sire effect, *A* is the additive numerator relationship matrix and *R* is the diagonal matrix of residual variances estimated at five levels. The levels of 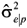, with p = 1,2,3,4,5 were grouped in EGs from -15 to -9, -8 to -3, -2 to +2, +3 to +8, and +9 to +15, respectively, for non-iterative models, and -20 to -12, -11 to -4, -3 to +3, +4 to +11, and +12 to +20, respectively, for iterative models. These groups were accommodated by identities matrices of appropriate order for each level.

Direct additive variance estimates in the random regression sire model were obtained by multiplying sire variance estimates by four ( 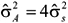). Residual variance estimates were obtained as the difference between phenotypic variance ( 
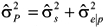
) and additive variance estimates ( 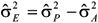). Expected breeding values (EBVs) were the double of expected progeny differences (EPDs), the latter being obtained from the sire model directly by the equation:

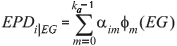


## Results

The distributions of animal weights along the environmental gradient in RRMg and RRMg-m (based on G450) were skewed slightly to the right (skewness of 0.15 and 0.16, respectively). Data distribution in RRMw and RRMw-m was less symmetric, with skewness of 0.67 and 0.70, resulting in the accumulation of records in EG = +15 (Figure 1a). When EGs were defined based on farm-year groups the records were concentrated in the central region of environmental gradient and led to a larger number of sires being excluded from the analysis compared to the farm-year-season-management groups (192 and 177 sires with 85,259 and 79,250 total records in RRMw and RRMg, and 220 and 242 sires with 89,784 and 90,735 total records in RRMw-m and RRMg-m and their iterative models, respectively).

Table 1 shows the parameter estimates of the model (with approximate standard errors for the Legendre polynomial coefficients and residual variances) in different analyses. In UM, there were only single estimates for residual variance and genetic variance. Hence, in Figure 2 and in the Supplementary Material, the variances are shown as lines to allow visual comparisons with RRMs (the lines are parallel to the environmental gradient axis).

Heritability estimates (h^2^) were higher in favorable and unfavorable environmental extremes (Figure 2). The minimal heritabilities were always in the middle-left region on the environmental gradient (EGs from -8 to -5 in non-iterative models and -13 to 0 in iterative models). We expected the curves to either increase or decrease (with the concavity facing out of the environmental gradient range) since linear (first degree polynomials) regression models were used. However, this was not observed. A change in the model altered the sharpness of the concavity and led to more variable estimates, as in RRMw, with h^2^ ranging from 0.19 (in EG = -6) to 0.29 (in EG = -15) and 0.42 (in EG = +15), or less variable estimates, as in RRMg-m, with h^2^ from 0.23 (in EG = -7) to 0.29 (in EG = -15) and 0.36 (in EG = +15). RRMg showed the lowest h^2^ estimates in unfavorable environments, but this situation was inverted in the favorable extreme, where the estimate was highest. The UM heritability estimate (h^2^ = 0.24) was lower than the RRM estimates along most of the environmental gradient, with larger differences in the favorable extreme. Different changes occurred when iterative models were applied to W450- and G450-based environmental variables. RRMITw-m and RRMw-m had a very similar shape, whereas RRMITg-m and RRMg-m showed important differences in extreme environments, with much higher heritabilities after iterations. Indeed, RRMITg-m had the highest heritabilities of all of the models.

Partitioning the estimates of residual variance into five levels based on a continuous additive genetic variance created abrupt leaps in the curves of residual and phenotypic variance estimates and indicated intense heteroscedasticity along EG levels. Phenotypic variance estimates ( 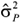) tended to increase along the environmental gradient as a whole and showed stable values within residual estimate classes. The additive genetic variance estimates ( 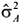) were greater at the extremes of the environmental gradient in all models. Residual variance estimates ( 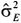) increased slightly along the environmental gradient but were variable within classes (they increased when p = 1, were stable when p = 2, and decreased when p = 3 to 5). The variance components estimates are shown in the Supplementary Material (Figures S1-S3).

RRMs estimated the covariance functions and displayed the genetic correlation estimates (r_g_) between environments as surface three-dimensional plots (Figure 3). The r_g_ were plotted on the z axis based on EG values for the x and y axes. This resulted in figures with "saddle" shapes in which r_g_ was minimal between opposite extremes (ranging from 0.08 in RRMw to 0.47 in RRMITw-m) and close or equal to one among similar environments in favorable or unfavorable regions. All of the models revealed a marked GEI between opposite extreme environments. The genetic correlation value of 0.8, which is indicative of a significant GEI (Robertson, 1959), separated the black part of the surface with less important GEI (r_g_ > 0.8) from the grey part with important GEI (r_g_ < 0.8). RRMg, RRMw-m and RRMITg-m yielded lower correlations between opposite extremes and had larger grey areas on the surface. RRMg-m had a higher r_g_ and smaller grey areas. RRMw and RRMITw-m were intermediate in their ability to identify GEI.

Adaptive reaction norms (ARN) were defined using predicted genetic values expressed as expected progeny differences (EPDs) along the environmental gradient. A sample of ARNs is shown in Figure S4. The ARN slopes indicate the angular coefficient of the sires' ordinary polynomials. These values were used in regression analyses to identify biases in the current selection programs. Regression analyses of the UM EPDs (constant and independent of environmental gradient) on RRM EPDs in EGs -15, zero and +15 in non-iterative models, and EGs -20, zero and +20 in iterative models, as well as on slopes, yielded significant results (p < 0.0001). The correlation between UM EPDs and favorable environment RRM EPDs (EG = +15) was positive and even greater (Table 2). The correlations between UM EPDs and ARN slopes ranged from 0.64 to 0.72.

The variance of the ARN slope is directly related to the importance of GEI and reflects the environmental sensitivity (Falconer, 1990), referred to as plasticity (in relation to larger absolute slopes) or robustness (in relation to smaller absolute slopes). Regression analyses for ARN slopes from different RRMs were consistent (p < 0.0001) and had coefficients of determination between 0.70 (RRMg X RRMg-w) and 0.98 (RRMw-m X RRMITw-m). Figure S5 shows the regressions and their equations and coefficients of determination.

## Discussion

The results described here show that different models generate consistent parameter estimates. The initial aim of using different environmental descriptors was to maximize the identification of GEI based on the concept that similarities between independent (EGs of W450 averages) and dependent (variance components and EPDs for W450) regression variables would lead to biases and lower significance of GEI. This occurred when comparing the RRMITw-m and RRMITg-m genetic correlation surfaces, but was not directly observed among non-iterative models or when heritabilities were considered. The low genetic correlation among extreme environments suggested that different groups of genes were being expressed. In agreement with Falconer (1960), we suggest that growth in low or high nutritional environments results in the differential expression of genes associated with growth and feed intake and efficiency. This affirmation, together with the results of the UM EPD regression analysis, indicates that current selection programs may be selecting for greater growth and feed intake, regardless of the feed efficiency. Environmental gradients, when defined by the CG averages, can generate connections among dependent and independent model variables that only can be explained by Wright's path analysis. This methodology is recommended by Lynch and Walsh (1997) for studies with related components in which correlations among indicators of latent (non-measurable) variables and the path coefficients are defined using structural equation models with simultaneous dependencies. Future work could examine the correlations and path coefficients for latent variables (gene group effects related to different trait components) in different environments. Such an analysis could help to explain differences in the importance of GEI and heritabilities in various RRMs since environmental descriptors generally correlate with the causal components of weight trait.

The importance of GEI in weight trait and the usefulness of the reaction norm concept as an effective model in this case need to be emphasized. Even so, choosing the best environment descriptor apparently depends on the desired breeding goal. Complex relationships among trait components are tied to the breeding goal and the model of choice can be indicated by larger genetic gains by generation for the chosen environments. With reaction norms, robustness and plasticity can be added as additional breeding goals to generate options for generalist or specialist sires.

In conclusion, we have demonstrated an important genotype-by-environment interaction in the 450-day weight trait of Nelore cattle analyzed by random regression reaction norm models using environmental variables defined by group averages. Genetic correlations were low between opposite extreme environments. These data indicate a significant re-ranking of sires in different environments and show the need to consider GEI effects, not only in large scale (across countries), but also within a national analysis. The UM EPDs showed a lower correlation with EPDs in unfavorable compared to intermediate and favorable environments, indicating that selection based on the predictions of UM genetic values is biased towards favorable environments.

Although the parameter estimates for the different models showed a joint variable tendency along the environmental gradient, changes in the environment descriptor interfered with these values. Iterative models amplified the distribution of data along the environmental gradient and yielded higher heritabilities. The use of G450-based environment descriptors altered the estimates of variance. This finding suggested the presence of intrinsic correlations with other genetic variables linked to physiological and morphological characters that make up the W450 trait. Such an association would explain the increase in heritability at unfavorable environmental extremes.

## Supplementary Material

The following online material is available for this article:

Figure S1 - Phenotypic variance estimates (in kg.kg) along environmental group (EG) in UM, RRMw, RRMg, RRMw-m and RRMw-g (a) and UM, RRMITw-m and RRMITg-m (b).
Figure S2 - Genetic additive variance estimates (in kg.kg) along environmental group (EG) in UM, RRMw, RRMg, RRMw-m and RRMw-g (a) and UM, RRMITw-m and RRMITg-m (b).
Figure S3 - Residual variance estimates (in kg.kg) along environmental group (EG) in UM, RRMw, RRMg, RRMw-m and RRMw-g (a) and UM, RRMITw-m and RRMITg-m (b).
Figure S4 - Adaptive reaction norms (ARNs) of “top 10 UM EPDs” sires, expressed in EPDs (in kg) plotted along the environmental gradient (EG) for different models (RRMw, RRMg, RRMw-m, RRMg-m, RRMITw-m and RRMITg-m).
Figure S5 - Regressions between 450-day weight ARN slopes estimated by different models (RRMw x RRMg, RRMw-m x RRMw, RRMg-w, RRMg x RRMg-m, RRMw-m x RRMg-m, RRMITw-m x RRMw-m and RRMITg-m x RRMITw-m), with their respective regression equations and regression coefficients (R^2^) (p < 0.0001 for all regressions).
This material is available as part of the online article from http://www.scielo.br/gmb.

## Figures and Tables

**Table 1:** Random regression sire variance estimates of the Legendre polynomial intercept (I, k = 1) and slope (S, k = 2), covariance (IxS) and residual variance estimates for different classes (p from 1 to 5) in different models (UM, RRMw, RRMg, RRMw-m, RRMw-g, RRMITw-m, RRMITg-m). The approximate standard errors are shown below each parameter.

	Intercept (I) (k = 1)	Slope (S) (k = 2)	I x S	${\hat{\sigma }}_{e|p=1}^{2}$	${\hat{\sigma }}_{e|p=2}^{2}$	${\hat{\sigma }}_{e|p=3}^{2}$	${\hat{\sigma }}_{e|p=4}^{2}$	${\hat{\sigma }}_{e|p=5}^{2}$
UM	80.6			629.2				
	5.6			6.9				
RRMw	66.9	19.3	14.4	478.1	562.4	590.3	664.0	738.84
	6.2	4.7	3.8	11.8	10.1	15.5	26.9	40.6
RRMg	72.0	18.3	16.6	530.1	575.5	636.6	628.4	762.8
	6.8	5.3	4.1	12.6	9.8	13.8	22.3	43.8
RRMw-m	71.9	14.5	12.8	479.4	553.8	614.9	657.1	763.7
	6.1	3.9	3.5	10.3	9.5	14.4	24.0	43.1
RRMg-m	81.9	11.2	9.6	523.2	592.8	621.9	630.3	750.5
	6.6	3.3	3.0	9.5	9.2	11.8	16.4	33.4
RRMITw-m	77.2	16.6	16.6	476.1	564.6	617.3	681.5	854.0
	6.9	4.4	4.6	9.4	9.2	16.1	29.4	55.6
RRMITg-m	81.3	12.5	20.8	483.6	575.6	604.2	671.8	839.0
	6.5	4.1	4.7	9.4	8.8	13.4	21.4	42.7

**Table 2:** Correlation coefficients for the linear regression between expected progeny differences (EPDs) from UM and other models at specific points in the environmental gradient (EG = -15, 0 and +15 for RRMw, RRMg, RRMw-m and RRMg-m, and EG = -20, 0 and +20 for RRMITw-m and RRMITg-m). Only sires with progeny weights that were used in the analyses were considered (p < 0.0001 for all regressions).

	RRMw					RRMg			
	EG(-15)	EG(0)	EG(+15)	Slope		EG(-15)	EG(0)	EG(+15)	Slope
UM	0.77	0.99	0.96	0.76		0.66	0.97	0.92	0.64
			
	RRMw-m					RRMg-m			
	EG(-15)	EG(0)	EG(+15)	Slope		EG(-15)	EG(0)	EG(+15)	Slope
UM	0.85	0.99	0.97	0.75		0.88	0.97	0.96	0.72
			
	RRMITw-m					RRMITg-m			
	EG(-15)	EG(0)	EG(+15)	Slope		EG(-15)	EG(0)	EG(+15)	Slope
UM	0.86	1.00	0.97	0.76		0.78	0.97	0.94	0.69
