# Supplementary material for: Genotype by environment interaction for 450-day weight of Nelore cattle analyzed by reaction norm models
Source: Genet Mol Biol. 2009 Jun 1;32(2):281–7. doi: 10.1590/S1415-47572009005000027 (PMC3036923; doi:10.1590/S1415-47572009005000027)
Supplement: Supplementary file 1 — Figure S1 - Phenotypic variance estimates (in kg.kg) along environmental group (EG) in UM, RRMw, RRMg, RRMw-m and RRMw-g (a) and UM, RRMITw-m and RRMITg-m (b). Figure S2 - Genetic additive variance estimates (in kg.kg) along environmental group (EG) in UM, RRMw, RRMg, RRMw-m and RRMw-g (a) and UM, RRMITw-m and RRMITg-m (b). Figure S3 - Residual variance estimates (in kg.kg) along environmental group (EG) in UM, RRMw, RRMg, RRMw-m and RRMw-g (a) and UM, RRMITw-m and RRMITg-m (b). Figure S4 - Adaptive reaction norms (ARNs) of “top 10 UM EPDs” sires, expressed in EPDs (in kg) plotted along the environmental gradient (EG) for different models (RRMw, RRMg, RRMw-m, RRMg-m, RRMITw-m and RRMITg-m). Figure S5 - Regressions between 450-day weight ARN slopes estimated by different models (RRMw x RRMg, RRMw-m x RRMw, RRMg-w, RRMg x RRMg-m, RRMw-m x RRMg-m, RRMITw-m x RRMw-m and RRMITg-m x RRMITw-m), with their respective regression equations and regression coefficients (R2) (p < 0.0001 for all regressions). This material is available as part of the online article from http://www.scielo.br/gmb. [file gmb-32-2-281-suppl1.pdf]

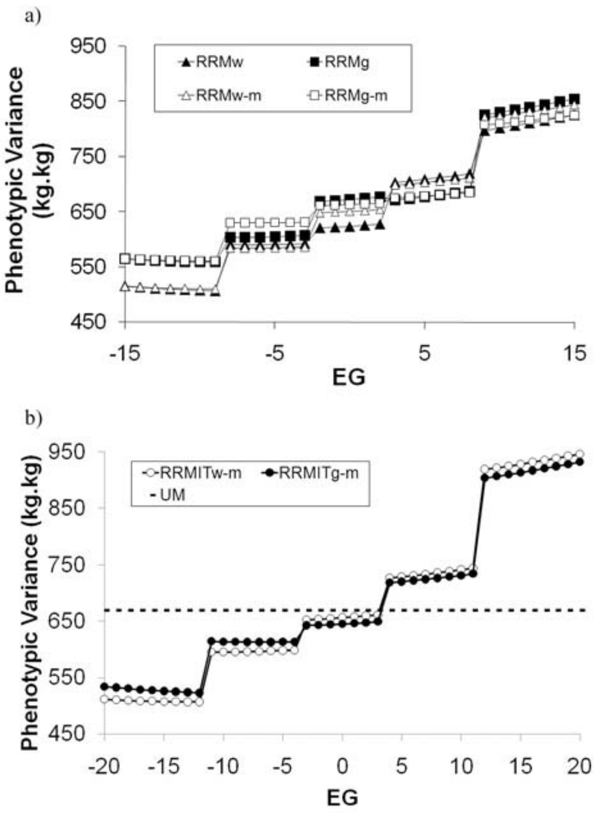

Figure S1 - Phenotypic variance estimates (in kg.kg) along environmental group (EG) in UM, RRMw, RRMg, RRMw-m and RRMw-g (a) and UM, RRMITw-m and RRMITg-m (b).

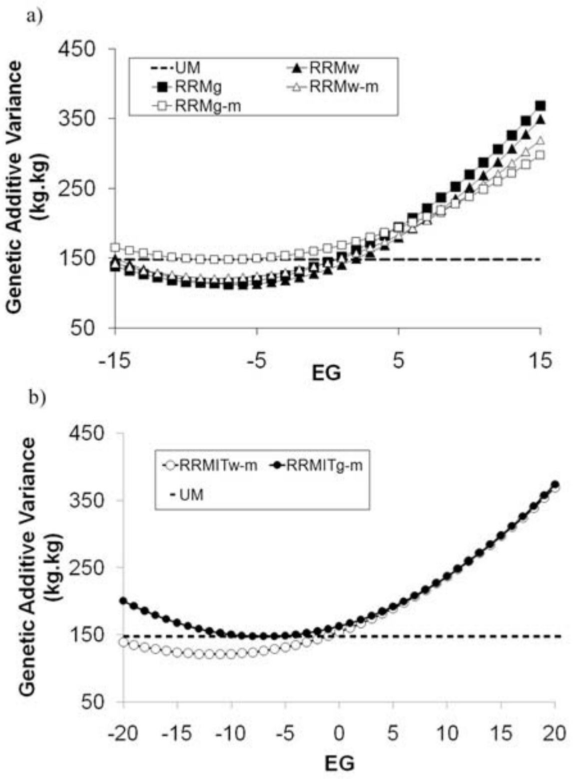

Figure S2 - Genetic additive variance estimates (in kg.kg) along environmental group (EG) in UM, RRMw, RRMg, RRMw-m and RRMw-g (a) and UM, RRMITw-m and RRMITg-m (b).

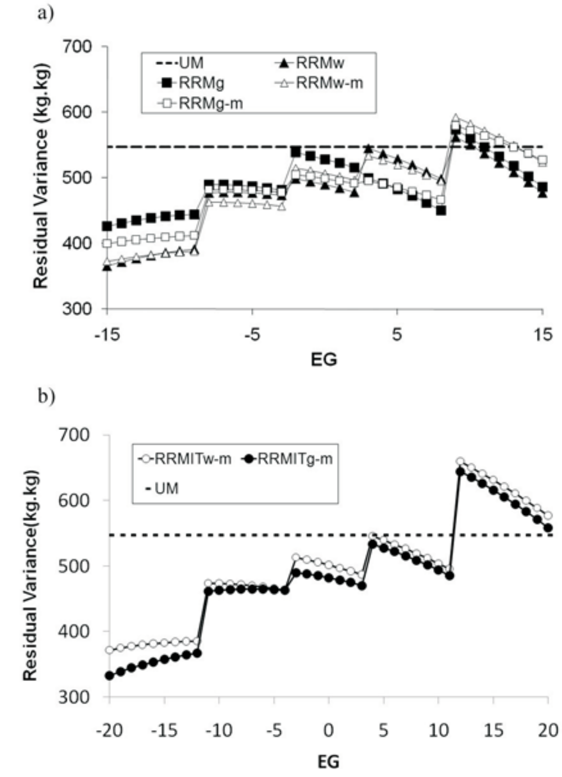

Figure S3 - Residual variance estimates (in kg.kg) along environmental group (EG) in UM, RRMw, RRMg, RRMw-m and RRMw-g (a) and UM, RRMITw-m and RRMITg-m (b).

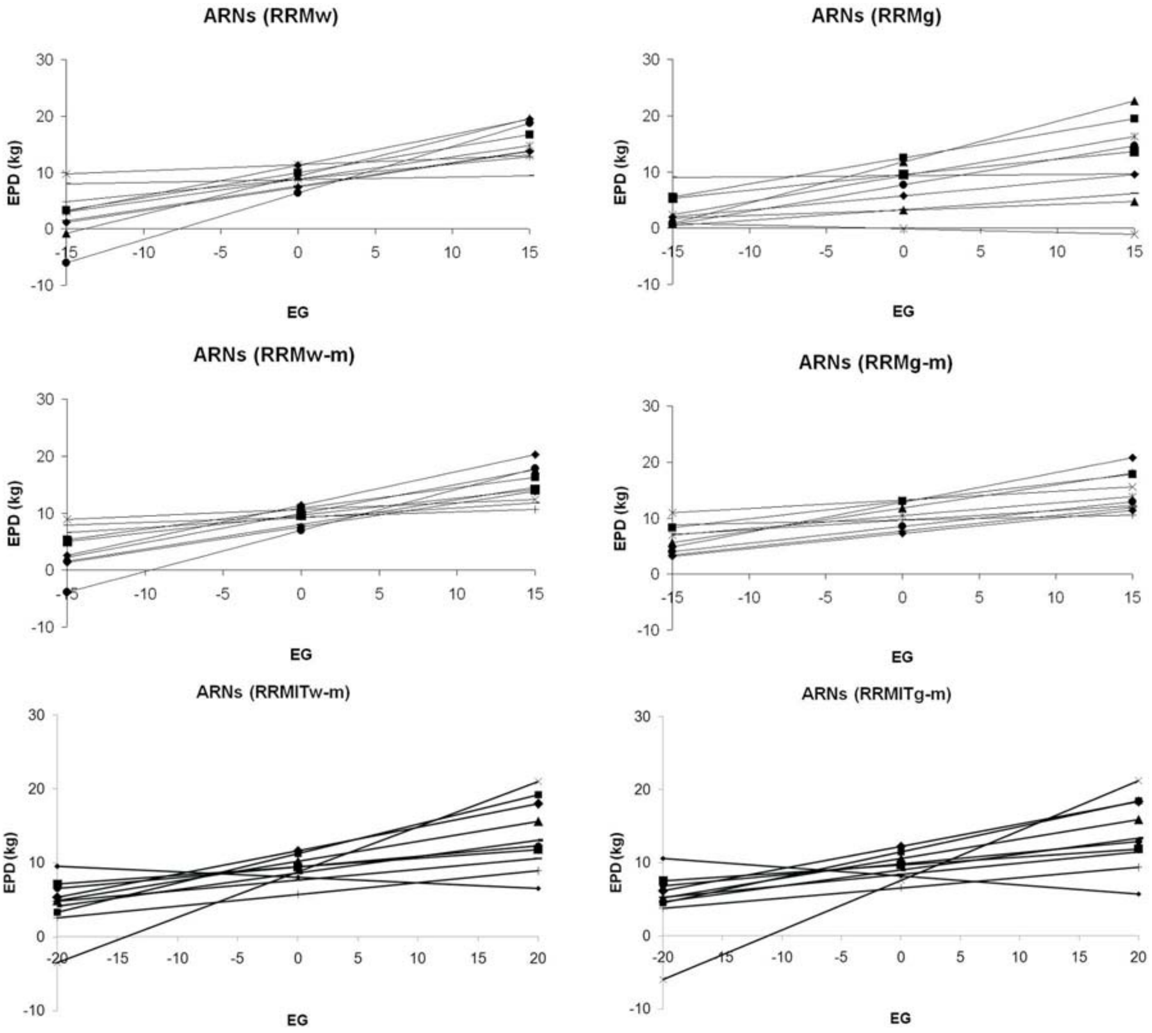

Figure S4 - Adaptive reaction norms (ARNs) of "top 10 UM EPDs" sires, expressed in EPDs (in kg) plotted along the environmental gradient (EG) for different models (RRMw, RRMg, RRMw-m, RRMg-m, RRMITw-m and RRMITg-m).

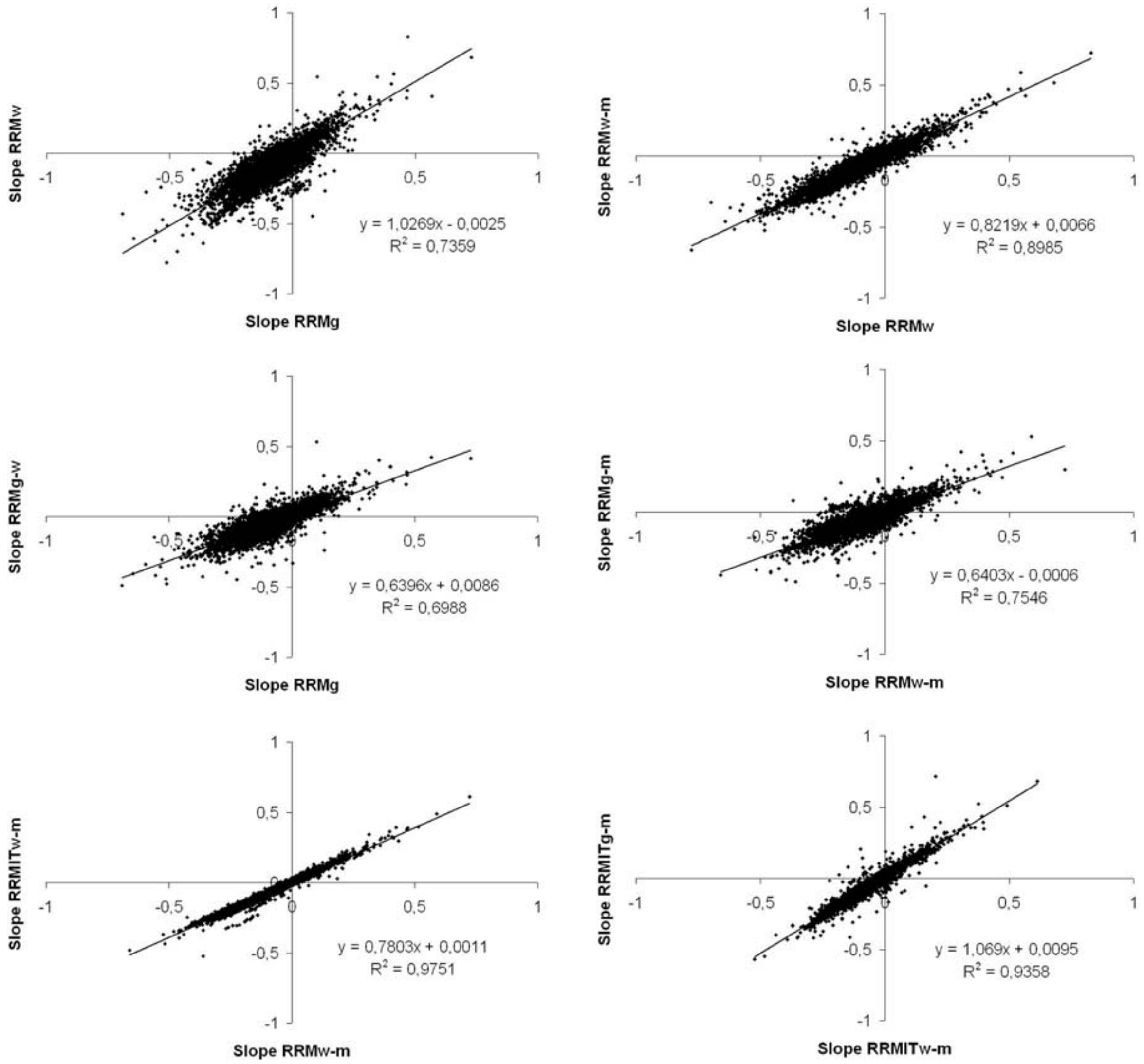

Figure S5 - Regressions between 450-day weight ARN slopes estimated by different models (RRMw x RRMg, RRMw-m x RRMw, RRMg-w, RRMg x RRMg-m, RRMw-m x RRMg-m, RRMITw-m x RRMw-m and RRMITg-m x RRMITw-m), with their respective regression equations and regression coefficients (R^2) ($p < 0.0001$ for all regressions).
